# The life and work of Dr. Fan Qingsheng: a pioneer in antibiotics research, agricultural microbiology, systems agriculture, and agricultural education in China

**DOI:** 10.1007/s13238-017-0374-x

**Published:** 2017-02-17

**Authors:** Jianping Xu

**Affiliations:** Department of Biology, McMaster University, Hamilton, ON L8S 4K1 Canada

**Figure 1:**
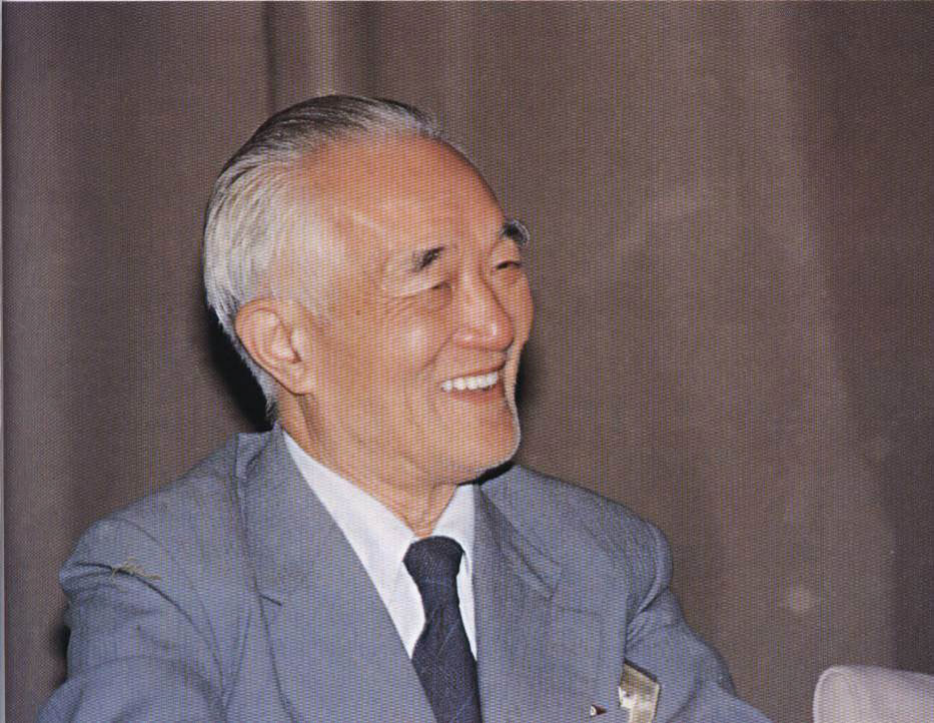
**Dr. Fan Qingsheng: a pioneer in antibiotics research, agricultural microbiology, systems agriculture, and agricultural education in China**

**Figure 2:**
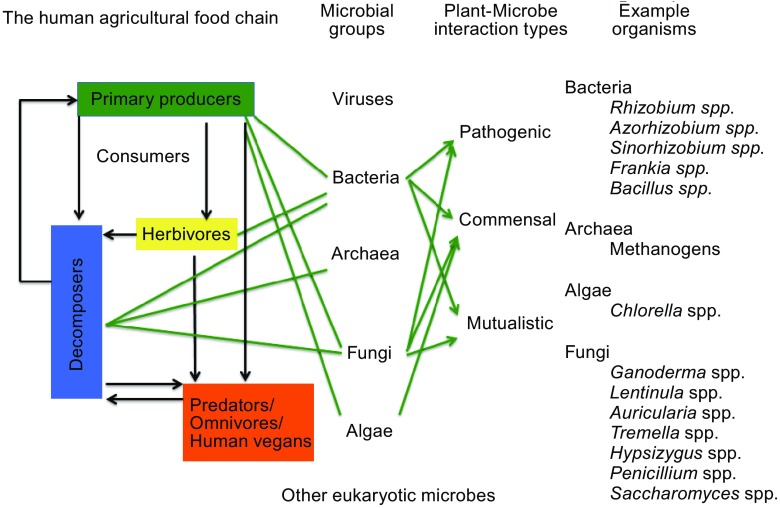
**A microbe-centric summary of Dr. Fan Qingsheng’s work on agricultural microbes and their roles in the broad systems agriculture framework**. Example organisms that Dr. Fan worked on are shown on the right. These microorganisms were investigated for their potential roles in enhancing crop productivity (e.g. *Rhizobia*), reducing diseases (e.g. *Penicillium* and *Streptomyces*), eliminating and/or bio-converting agricultural wastes into valuable goods (e.g. methanogens and edible fungi), and enhancing soil fertility (legumes and *Rhizobia*)

Microbes are everywhere in the Earth’s biosphere—they play crucial roles in agriculture, nutrient cycling, environmental protection, and animal and human health. However, despite the importance of microbes throughout human history, their roles remain to be fully recognized. In China, one of the key scientists who led to the recognition and utilization of microbes was Dr. Fan Qingsheng (樊庆笙博士, Fig. 1). Dr. Fan not only contributed significantly to the production of penicillin in China but also established the broad framework for the effective use of microbes to develop sustainable agriculture by increasing agricultural production, creating valuable goods from agricultural wastes, cleaning up pollutants in aquatic and soil environments, and enhancing soil fertility through organic farming. Indeed, he was a visionary both in establishing the theoretical framework of a microbe-centric sustainable agriculture and in demonstrating the effectiveness of this approach through over 60 years of tireless work on a diversity of organisms across many ecological niches (Fig. 2).

**Figure 3:**
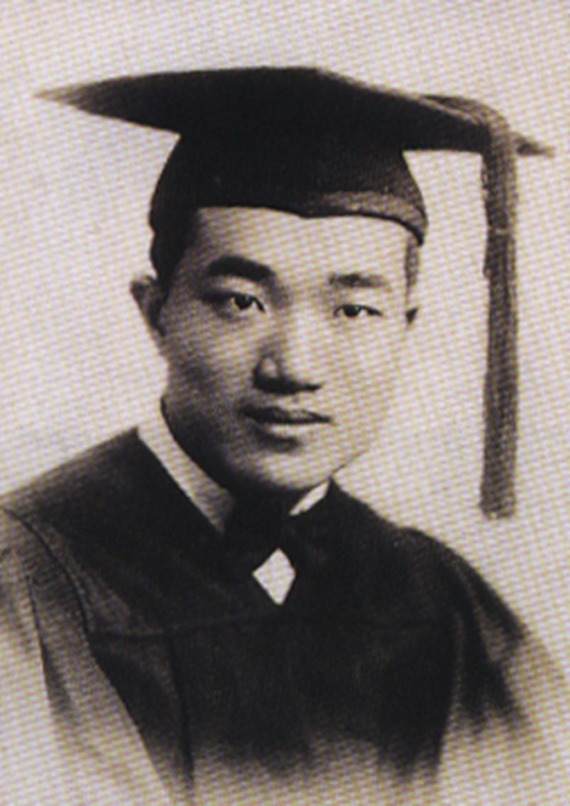
**Graduating from Jinling University with an honor BSc in Forestry in 1933**

Dr. Fan was born on August 4, 1911, in the township of Xizhoushi, Changshu county, Jiangsu province, along the south shore of the Yangtze River, northwest of Shanghai. The oldest of nine children in the family, Dr. Fan spent much of his childhood in the countryside where he watched fishermen fishing along the Yangtze River, observed how crops, vegetables, and trees were grown in the traditional Chinese agricultural system, and helped his family obtain food (Fan, 2011). His passion for agriculture started during his youth when he witnessed the devastating effects of hunger, malnutrition, and diseases on the community. He believed that there must be ways to improve agriculture and alleviate the suffering. Like in most Chinese communities, even during politically unstable times, providing the best possible education for their children was a top priority for many families, including Dr. Fan’s. Dr. Fan seized learning opportunities, earning some of the top grades in his classes throughout his elementary, middle, and high school years. Upon graduating from Cuiying High School in Suzhou, he was admitted to the Department of Forestry, Jinling University in Nanjing in 1929. Because of the lack of financial resources, his family had to borrow money to cover his first-year’s tuition at Jinling University. However, due to his excellent academic results, he was awarded full scholarships from the second to the fourth year of his study, including being hired as a teaching assistant to help organize laboratory classes—an extremely rare opportunity for undergraduate students. His top academic performance earned him the “Golden Key” award, the highest honor bestowed by Jinling University for its graduating students, in 1933 (Fan 2011; Fig. 3). Right after graduation, he was offered an assistant lecturer position for classes in Botany and Plant Taxonomy. In this role, he helped establish the largest herbarium in China at the time (Li, 2003). His solid training in botany during his undergraduate years played a vital role in developing his framework on the diversity of plant-microbe interactions and their importance in agriculture.

**Figure 4:**
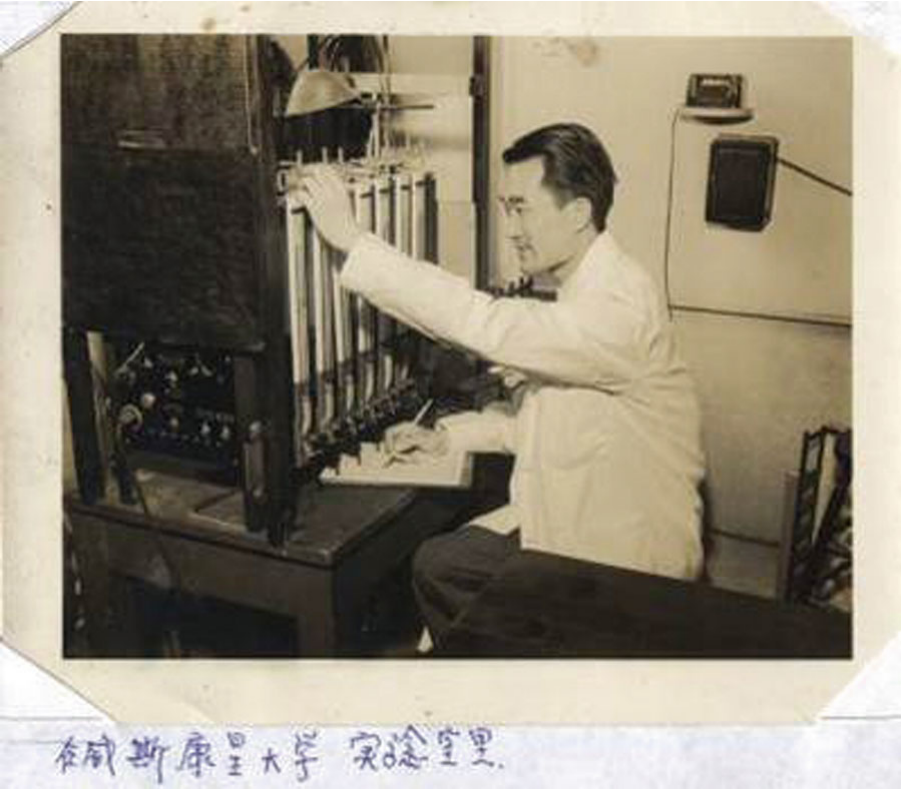
**Working with a gas exchange testing machine at the University of Wisconsin-Madison for his PhD degree**

In 1937, the Japanese invasion caused many government agencies across eastern China, including universities, to move to western China. Jinling University was relocated to Huaxiba district in Chengdu, Sichuan province. While there, Dr. Fan was promoted to Lecturer and continued to teach Botany (Li, 2003). In the summer of 1940, the Rockefeller Foundation offered one full graduate scholarship to a junior staff member in the Faculty of Agriculture at Jinling University to study at an American university. However, instead of sending one person for three years to study for a PhD degree, the Faculty of Agriculture decided, with permission from the Foundation, to make the best use of the opportunity and used the funding to send three young scholars to the US for one year study each to do their MSc degrees. Dr. Fan was among those three young scholars and was admitted to the Department of Botany in the Faculty of Agriculture at the University of Wisconsin-Madison. However, after obtaining his MSc in one year of study in 1941, his excellent academic performance and aptitude for learning caught the attention of the microbiologist Dr. W.W. Umbreit who suggested that he worked on his PhD degree on the physiology of photosynthesis in the alga *Chlorella pyrenoidosa* (Fig. 4). Dr. Fan successfully completed his PhD studies in 1943 and published two papers from his thesis in the Journal of General Physiology (Fan et al., 1943) and Journal of Bacteriology (Fan and Umbreit, 1943).

During his three years of study at the UW-Madison, Dr. Fan devoted all his time to research and to learning the diverse aspects of microbiology. At that time, the Department of Agricultural Bacteriology had two world-leading research programs, one on symbiotic nitrogen fixation between *Rhizobia* bacteria and legumes and the second on the biochemistry of antibiotics, especially on the development of *Penicillium* strains capable of producing high quantities of penicillin, the first antibiotic (https://bact.wisc.edu/history_contributions.php). Dr. Fan learned as much as he could about both frontiers. Due to the ongoing Second World War (WWII), the production of penicillin especially attracted his attention. Since its discovery by Alexander Fleming in 1929, this antibiotic has saved millions of lives from infectious diseases, most prominently the injured soldiers at the front lines in Europe. During WWII, tens of millions of Chinese were dying each year from infectious diseases (Watts, 2013). Dr. Fan believed penicillin could similarly save many lives in China. However, because of the difficulty of returning to China due to the ongoing WWII across the Asian-Pacific region, Dr. Fan decided to use his microbiology knowledge and worked at Seagram & Sons, Inc. in Louisville, Kentucky on fermentation technologies while searching for an opportunity to return to China. As we will see below, these three areas of microbiology (penicillin, microbial fermentation, and biological nitrogen fixation) all became important topics of Dr. Fan’s research after his return to China.

**Figure 5:**
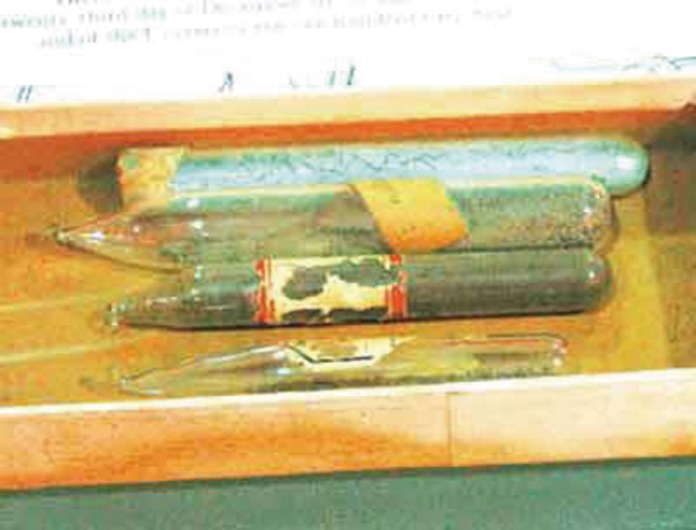
**Three strains of** ***Penicillium*** **brought by Dr. Fan from the US to China in 1944**. These strains were critical for starting the production of penicillin in China. The strains are now on display at the Chinese Agricultural Museum in Nanjing Agricultural University

The opportunity to return to China came in late 1943 when the American Bureau for Medical Advancement in China (ABMAC) decided to help China establish its first blood bank in Kunming, including providing all the required equipment, personnel, and technology, to help the Allied countries fight against Japanese aggression in the Asian-Pacific Region (Watt, 2013). Dr. Fan successfully obtained the position of diagnostic bacteriologist on the team, with the responsibility of ensuring the safety of blood and blood products. He also suggested to ABMAC that he would like to help produce penicillin in China. The suggestion was accepted and ABMAC helped procure the necessary strains (three strains total; Fig. 5), materials and equipment for growing the fungi and for isolating, purifying, and testing the antibiotic. After finished their training, a team of eight experts and all their equipment and supplies left New York City on January 20, 1944 on a long and difficult journey. They sailed through the western Atlantic Ocean, the Caribbean Sea, the Panama Cannel, the southern Pacific Ocean around New Zealand and Australia, the Indian Ocean, and finally landed in Bombay, India. They then travelled by train from Bombay to Calcutta and finally to Ledo in Assam where they were air-lifted by a military transport plane over “The Hump”—the eastern Himalayan Mountains—to Kunming in June 1944 (Li, 2003; Fan, 2011).

While in Kunming, Dr. Fan was in charge of diagnosis for the blood bank, ensuring the safety of blood and blood products for soldiers fighting in the China-India-Burma Theater of war against the Japanese invaders. In addition, he joined hands with Zhu Jiming and Dr. Tang Feifan, a prominent virologist and director of the Chinese Center for Disease Control and Prevention at the time, in penicillin research. They successfully produced the first batch of penicillin, making China the seventh country capable of producing this antibiotic (Fan 2011). Their work helped save tens of thousands of lives from 1944 to 1946 and contributed to subsequent rapid medical developments in both blood transfusion and antibiotic research and production in China.

After the end of WWII, Dr. Fan returned to Jinling University to teach “Microbial Physiology” and was promoted to Professor in June 1946. Aside from his regular academic responsibilities at Jinling University in Nanjing, he travelled regularly between Nanjing and Shanghai, supervising *Penicillium* strain improvement and working with Dr. Tong Cun and others on the production of penicillin at the Biochemical Products Research Laboratory of the Chinese National Institutes of Health in Shanghai. Their work successfully led to the large-scale industrial production of medical-grade penicillin. Dr. Fan translated “penicillin” to “青霉素” in Chinese, now a household word in China (Fan, 2011). Aside from penicillin, in the 1980s and 1990s, he also led the development of antibiotics to control agricultural pests and pathogens (e.g. Sheng et al., 1983; Li et al. 1993).

After returning to the Faculty of Agriculture at Jinling University, Dr. Fan began his systematic investigations on soil microbes and biological nitrogen fixation, especially on the effects of symbiotic nitrogen-fixing bacteria on legumes and on soil fertility in general (Cao and Fan, 1957; Fan, 1963). He and his team isolated hundreds of *Rhizobia* strains from soybeans, peanuts, peas, and Chinese milk vetch plants. They conducted numerous cross-inoculation experiments to test for host specificity and symbiotic nitrogen fixation efficiency. Their work contributed to our understanding of soil microbiology and the development of several highly successful products for agricultural application that have led to significant increases in both crop productivity and soil fertility (Fan, 1985; 1986).

**Figure 6:**
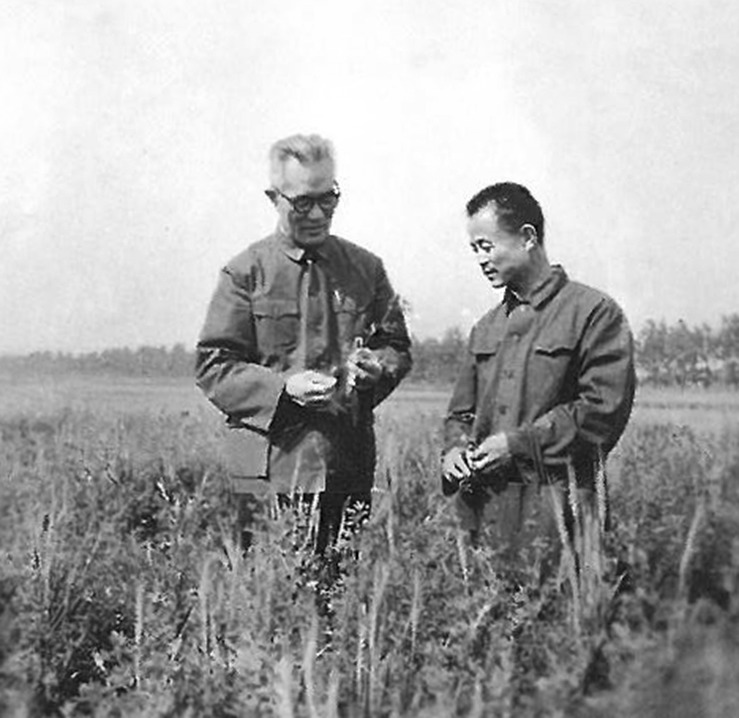
**Dr. Fan in a field of** ***Astragalus sinicus***

Among the products and technologies associated with Dr. Fan’s research on biological nitrogen fixation, the most notable was probably the improved productivity and range expansion of the Chinese milk vetch *Astragalus sinicus*, “紫云英” in Chinese (Fan et al., 1987). *Astragalus* is among the largest genera of flowering plants with different species having different ranges and distributed in different parts of the world. These leguminous plants can not only fix nitrogen and enrich soil fertility but also serve as animal feed, source of pollen for bees for honey production, and herbal medicine across the globe, among other uses. *A. sinicus* is naturally distributed in southern China, primarily south of the Yangtze River, and farmers have been growing it in winter months as a source of “green manure” for rice fields for centuries. However, farmers in central and northern China had not been able to enjoy this benefit, despite repeated introduction trials. Dr. Fan and his colleagues hypothesized that the lack of appropriate nitrogen fixing bacteria in the soil was the cause of the failed introduction. From 1958 to mid-1970s, Dr. Fan was not permitted to teach or conduct research at his university, and was sent to work as a farmer in the countryside (Fan, 2011; Li, 2003). However, he used the opportunity to test his hypothesis and through trial and error, he and his colleagues successfully selected appropriate *Rhizobia*-*A. sinicus* combinations that allowed range expansion of *A. sinicus* all the way to the Yellow River basin in northern China (Fan et al., 1987; Li, 2003; Fig. 6). This work has contributed enormously to agricultural sustainability in central and northern China and won him the First Chinese Science Congress’s 1st Prize in 1978. With the restoration of his teaching and research responsibilities after the “Cultural Revolution” in the late 1970s at Nanjing Agricultural University, Dr. Fan and his group continued to investigate the ecology, physiology, biochemistry, and genetics of nitrogen-fixing bacteria and contributed to further increases in legume production and soil fertility in China (e.g. Fan, 1986; Fan and Rao, 1986; Li, 2003).

**Figure 7:**
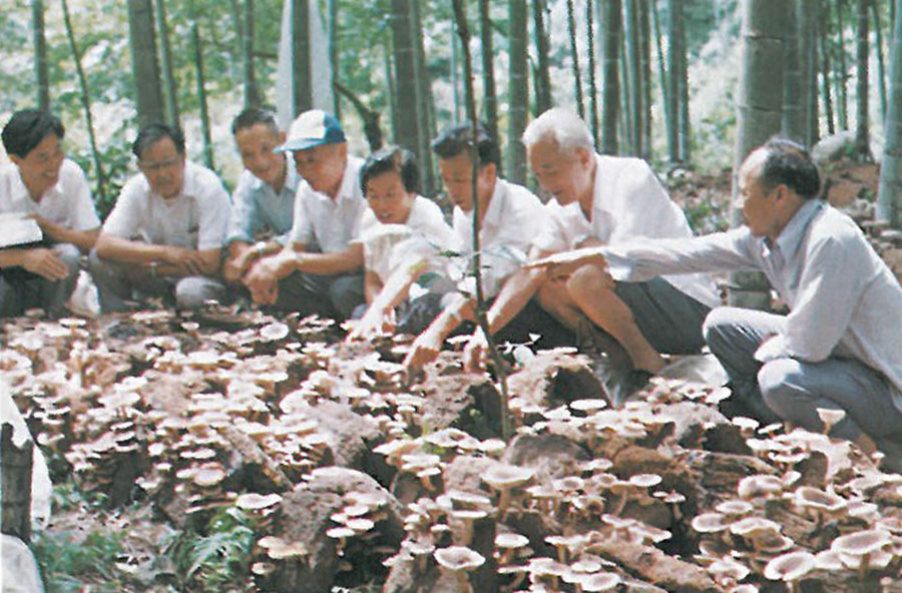
**Dr. Fan at a mushroom fruiting facility**

Productivity in ecosystems is often assessed by the amount of biomass production. However, only a small proportion of the biomass produced in agriculture, forestry, and animal husbandry is directly used or consumed by humans. The remaining biomass is largely under-utilized, completely wasted, or even becomes a source of environmental pollution. Dr. Fan saw such wasted biomass as a potential resource that could be converted to valuable goods through microbial action. To achieve this goal, he aggressively pushed for broad investigations on domesticating and cultivating edible and medicinal mushrooms using waste biomass since the late 1970s (e.g. Li et al. 1991; Fig. 7). His vision for mushroom production was far-reaching. China now is the No. 1 producer of edible and medicinal mushrooms in the world, accounting for about 70% of the world’s total mushroom production and with a cultivated species diversity far exceeding those in other countries. In addition, he and his team established the first anaerobic microbiology facility in China (Wang et al., 1984; Li, 2003) and initiated a series of studies that helped achieve several microbe-centric innovations in biofuel production, the elimination of plant pathogens and pests, and the degradation of organic pollutants from both agricultural and industrial runoff (e.g. Li et al., 1993; Li, 2003). The processed solid wastes were further used as fertilizers on agricultural fields, creating an extremely beneficial positive feedback loop. These areas of research by Dr. Fan showed that productive, healthy, and sustainable agricultural ecosystems were possible in China. The production of edible mushrooms serve as an additional source of income for farmers and an excellent source of nutrients; the production of biofuels such as methane gas also helps minimize energy costs and environmental pollution; and the spent mushroom substrates and solid wastes from anaerobic digesters further enrich soil fertility (Li, 2003).

Dr. Fan tirelessly advocated and tested the theoretic framework of a microbe-centric systems approach for sustainable agricultural and forestry management (Li, 2003). In this approach, invisible microbes play multiple critical roles. For example, in crop production, he believed that a productive and sustainable agriculture system required cost-effective ways to maintain soil fertility. He demonstrated that this could be achieved by creating conditions that (i) favor the growth of nitrogen-fixing microorganisms (e.g. by growing legumes), resulting in increased levels of nitrogen in the soil; (ii) facilitate colonization by mycorrhizal fungi (to increase the level of soluable phosphorus in the soil; and (iii) return processed and spent biomass (e.g. from anaerobic digestion and mushroom growing) to agricultural fields to increase the levels of organic matter, potassium, and other elements. He also showed that a healthy soil microbial community containing various beneficial microbes could help plants defend against infectious diseases and agricultural pests. He suggested that a similar microbe-centric systems approach could be applied to forestry, animal husbandry, and fisheries. Indeed, his broad vision was to link all the components in the human food chain together, in a holistic way and through microbial actions, to make the best use of the biomass generated by the primary producers: plants on land and algae in aquatic environments (Fig. 2). Through his own work, he has demonstrated the feasibility for many of the key components in this ecosystems approach to the improvement of agriculture.

Aside from being a visionary in agronomy and a pioneer in agricultural microbiology, Dr. Fan was also an excellent educator as evidenced by over 60 years of contribution to the training of tens of thousands of undergraduate students, graduate students, postdoctoral fellows, visiting scientists, and applied technical workers on the front lines of agriculture. He taught a diversity of courses including Botany, Plant Taxonomy, Plant Pathology, Soil Microbiology, Bacteriology, Agricultural Microbiology, Microbial Physiology, Microbial Taxonomy, and the Microbiology of Nitrogen Fixation. He also organized many workshops. His impacts were not limited to those who directly studied and/or worked with him, but extended to those from across China and outside of China. The workshops that Dr. Fan organized included the following three very timely and important ones that helped re-launch microbiology education in the Chinese agricultural education sector. The first was the month-long Microbiology Training Workshop in 1980 involving over 100 teachers from agricultural universities and colleges across China. This was the first national workshop for training microbiology teachers after the Chinese higher education system was re-instated following “Culture Revolution”. Expert microbiologists from across China were invited to give presentations on a broad diversity of topics in microbiology. An edited book called *Advances in Microbiology* was subsequently published and used as a reference by teachers (Fan and Chen, 1984). The second was the month-long National Anaerobic Microbiology Workshop in 1980. Among the speakers at this workshop was the then-President of the American Society for Microbiology Dr. R. H. Hungate. This workshop helped start anaerobic microbiology research in China. The third was a one-year training program in Microbial Genetics and Biotechnology in 1985–1986 for graduate students and young researchers from all major agricultural universities, with lectures by invited experts from China, Japan, and the US. These workshops played a vital role in training microbiologists and molecular biologists in the agricultural university system across China. The impact of these workshops can still be felt even today.

In his own teaching and lecturing, Dr. Fan’s presentations were always well prepared, logical, vivid, concise, and updated. Whenever possible, he linked real issues in agriculture to the key concepts in his lectures. In his teaching, he stressed the importance of the evidence-based approach to scientific discovery. While such an approach is essential in science, it has not always been accepted. For example, from the late 1970s to mid-1980s, an outspoken “citizen scientist” claimed that, using biological magnetism, he was capable of changing the genetic makeup of plants and inducing nitrogen-fixing root nodules in many non-leguminous crops such as wheat, rice, and cotton (Wang, 1981). His extraordinary claim attracted widespread attention from journalists working for a diversity of national and local newspapers and magazines. Many people, including some scientists, jumped to their feet to applaud such an “extraordinary invention” (e.g. Su, 1981; Wu, 1987). However, extraordinary claims require extraordinary evidence. As one of the most authoritative figures on symbiotic nitrogen fixation in China, Dr. Fan was asked to support such claims. He stood firm in his request for evidence of nitrogen fixation and was met with a smear campaign. Fortunately, science eventually triumphed in this case in Dr. Fan’s favor, demonstrating that those “nodules” were caused by soil nematodes and unable to fix nitrogen (Fan, 2011; Wu, 1987; Li, 2003). This case played a big role in affirming the importance of an “evidence-based approach” in agricultural and other types of scientific research in China.

Dr. Fan’s framework of microbe-based, systems view of agriculture is also evident in the textbooks and opinion pieces that he wrote (Qing, 2011). He authored or co-authored the general textbooks *Agricultural Microbiology*, *Microbiology*, and *Soil Microbiology*, as well as the specialized books *Microbiology of Nitrogen-Fixation*, *Advances in Microbiology*, and *Microbial Physiology*. He was the Editor-In-Chief for the *Biology* volume of the *Encyclopedia of Chinese Agriculture* book series. The book *Agricultural Microbiology* that Dr. Chen Huakui and he wrote has served as the standard textbook in the field since the 1950s and is still widely used today. Its fourth edition won the Chinese National Textbook Prize in 1989 (Chen and Fan, 1989).

Aside from his contributions to teaching and research, Dr. Fan was also an excellent administrator and an influential leader in a diversity of institutions and scholarly societies (Li, 2003). Among the many posts he held, he was a highly respected Provost of Jinling University (1951–1952) and President of Nanjing Agricultural University (1981–1984) during their critical transition periods. He served as the President of the International *Astragalus* Society and the Chinese Society of Mushroom Sciences. He chaired several national and international conferences on *Astragalus* research and Soil Microbiology. To help develop the Chinese mushroom industry, he organized two large international conferences on mushroom biology and cultivation: (i) the First International Symposium on Mushroom Biotechnology in 1989 in Nanjing; and (ii) the International Symposium on Science and Cultivation of Mushrooms in 1998. In the last few years of his life, he chaired the foundation in charge of establishing Jinling Research Institute within Nanjing Agricultural University.

Dr. Fan passed away in 1998, ending a long and decorative life and leaving a legacy that would have a lasting impact. Articles about him and his work continue to appear in books (e.g. Fan and Huang, 2013), newspapers, magazines, and the new media such as WeChat. Through over six decades of research and teaching, Dr. Fan left a large footprint on antibiotics production, agricultural education, microbiology, and agricultural science in China. Several of his perspectives are more relevant now than ever. For example, his emphasis on a microbe-centric systems approach to agriculture has been gaining increasing attention and support in this era of genomics and metagenomics where microbes are found to play crucial roles in all ecosystems in Earth’s biosphere. His ideal of an agricultural system utilizing “green manure” and involving holistic biomass re-utilization and recycling should be emphasized in the face of rapid environmental degradation in our cities and rural communities. Similarly, his insistence on evidence-based science should serve as a constant reminder in our pursuit of knowledge, regardless of the discipline.
